# Supplementary material for: A Random Matrix Approach to Credit Risk
Source: PLoS One. 2014 May 22;9(5):e98030. doi: 10.1371/journal.pone.0098030 (PMC4031172; doi:10.1371/journal.pone.0098030)
Supplement: Appendix S1 — (PDF) [file pone.0098030.s001.pdf]

Appendix S1 Identifying a Gaussian integral in the average distribution of asset values

$$\langle p^{(\mathrm{mv})}(V)\rangle = \int p^{(\mathrm{corr})}(W)p^{(\mathrm{mv})}(V,SWW^\dagger S)\mathrm{d}[W] \tag{41}$$

$$= \left(\frac{1}{\sqrt{2\pi}}\right)^{2K+KN}\sqrt{N}^{KN}\int \exp\left(-\frac{N}{2}\mathrm{tr}WW^\dagger\right) \times \int \exp\left(-i\omega^\dagger V\right)\exp\left(-\frac{T}{2}\omega^\dagger SWW^\dagger S\omega\right)\mathrm{d}[\omega]\mathrm{d}[W] \tag{42}$$

$$= \left(\frac{1}{\sqrt{2\pi}}\right)^{2K+KN}\sqrt{N}^{KN}\int \exp\left(-i\omega^\dagger V\right) \times \int \exp\left(-\frac{N}{2}\mathrm{tr}WW^\dagger\right) \times \exp\Big(-\frac{T}{2}\omega^\dagger SWW^\dagger S\omega\Big)\mathrm{d}[W]\mathrm{d}[\omega] \tag{43}$$

In the last steps, we took advantage of the fact that the term $\omega^\dagger SWW^\dagger S\omega$ is a scalar, which can evidently be written as trace. As the trace is invariant under cyclic permutation, we can express this term as $\mathrm{tr}(WW^\dagger S\omega\omega^\dagger S)$. Hence, we write

$$\langle p^{(\mathrm{mv})}(V)\rangle = \left(\frac{1}{\sqrt{2\pi}}\right)^{2K+KN}\sqrt{N}^{KN}\int \exp\left(-i\omega^\dagger V\right) \times \int \exp\left(-\frac{1}{2}\mathrm{tr}(WW^\dagger(NI+TS\omega\omega^\dagger S))\right)\mathrm{d}[W]\mathrm{d}[\omega] \tag{44}$$

$$= \left(\frac{1}{\sqrt{2\pi}}\right)^{2K+KN}\sqrt{N}^{KN}\int \exp\left(-i\omega^\dagger V\right) \times \int \exp\left(-\frac{1}{2}\sum_{n=1}^{N}(w_n^\dagger(NI+TS\omega\omega^\dagger S)w_n)\right)\mathrm{d}[w_n]\mathrm{d}[\omega] \tag{45}$$

$$= \left(\frac{1}{\sqrt{2\pi}}\right)^{2K+KN}\sqrt{N}^{KN}\int \exp\left(-i\omega^\dagger V\right) \times\left(\int \exp\left(-\frac{1}{2}w^\dagger(NI+TS\omega\omega^\dagger S)w\right)\mathrm{d}[w]\right)^N \mathrm{d}[\omega] \tag{46}$$

where I is the unit matrix. The last step can be accomplished, as the components of W are independent identically distributed, hence we can denote the n-th column vector of W, w_n by w. Thus, we can simplify the integration over the matrix W to the integration over the vector $w \in \mathbb{R}^K$ to the power of N. The integral over $\mathrm{d}[w]$ is simply a Gaussian integral, as indicated by x_i, x_j and A_{ij}. As w_n consists of K components, this gives an additional factor $\sqrt{2\pi}^{KN}$ and thus leads to

$$\langle p^{(\mathrm{mv})}(V)\rangle = \frac{\sqrt{N}^{NK}}{(2\pi)^K}\int \exp\left(-i\omega^\dagger V\right)\frac{1}{\sqrt{\det(NI+TS\omega\omega^\dagger S)}^N}\mathrm{d}[\omega] \tag{47}$$
