# Supplementary material for: A Random Matrix Approach to Credit Risk
Source: PLoS One. 2014 May 22;9(5):e98030. doi: 10.1371/journal.pone.0098030 (PMC4031172; doi:10.1371/journal.pone.0098030)
Supplement: Appendix S2 — (PDF) [file pone.0098030.s002.pdf]

Appendix S2 Derivation of component-wise average distribution of asset values

$$
\begin{aligned}
\langle p^{(\mathrm{mv})}(V)\rangle &= \frac{1}{(2\pi)^K}\frac{1}{\Gamma(N/2)}\int \exp\left(-i\omega^\dagger V\right) \\
&\quad\times \int_0^\infty z^{\frac{N}{2}-1}\exp\left(-(1+\frac{T}{N}\omega^\dagger SS\omega)z\right)\mathrm{d}z\,\mathrm{d}[\omega] && (48)\\
&= \frac{1}{(2\pi)^K}\frac{1}{\Gamma(N/2)}\int_0^\infty z^{\frac{N}{2}-1}\exp\left(-z\right) \\
&\quad\times \int \exp\left(-i\omega^\dagger V\right)\exp\left(-\frac{T}{N}\sum_{k=1}^{K}\sigma_k^2\omega_k^2 z\right)\mathrm{d}[\omega]\mathrm{d}z && (49)\\
&= \frac{1}{(2\pi)^K}\frac{1}{\Gamma(N/2)}\int_0^\infty z^{\frac{N}{2}-1}\exp\left(-z\right) \\
&\quad\times \prod_{k=1}^{K}\left[\int \exp\left(-i\omega_k V_k\right)\exp\left(-\frac{T}{N}\sigma_k^2\omega_k^2 z\right)\mathrm{d}\omega_k\right]\mathrm{d}z && (50)\\
&= \frac{1}{(2\pi)^K}\frac{1}{\Gamma(N/2)}\int_0^\infty z^{\frac{N}{2}-1}\exp\left(-z\right) \\
&\quad\times \prod_{k=1}^{K}\left[\frac{\sqrt{\pi N}}{\sqrt{zT}\sigma_k}\exp\left(-\frac{NV_k^2}{4Tz\sigma_k^2}\right)\right]\mathrm{d}z && (51)\\
&= \frac{1}{(2\pi)^K}\frac{1}{\Gamma(N/2)}\left(\prod_{k=1}^{K}\frac{1}{\sigma_k}\right) \\
&\quad\times \int_0^\infty z^{\frac{N}{2}-1}\exp\left(-z\right)\left(\frac{\pi N}{zT}\right)^{\frac{K}{2}}\exp\left(-\frac{N}{4Tz}\sum_{k=1}^{K}\frac{V_k^2}{\sigma_k^2}\right)\mathrm{d}z && (52)
\end{aligned}
$$
