# Supplementary material for: A Random Matrix Approach to Credit Risk
Source: PLoS One. 2014 May 22;9(5):e98030. doi: 10.1371/journal.pone.0098030 (PMC4031172; doi:10.1371/journal.pone.0098030)
Supplement: Appendix S3 — (PDF) [file pone.0098030.s003.pdf]

Appendix S3 Normalization of the average distribution of asset values

We consider the variance of the i-th asset value V_i,

$$\mathrm{var}(V_i) = \int \mathrm{d}[V]\ V_i^2 \langle p^{(\mathrm{mv})}(V)\rangle \tag{53}$$

We can solve this integral by using hyperspherical coordinates.

$$\rho \equiv \sqrt{\sum_{k=1}^{K} \frac{V_k^2}{\sigma_k^2}} \qquad \text{with} \qquad \frac{V_i}{\sigma_i} \equiv \rho \cos\vartheta \tag{54}$$

for a chosen component V_i/σ_i. Now we can write the integral in Eq. (53) as

$$\begin{aligned}\mathrm{var}(V_i) &= \sqrt{\frac{N}{2\pi T}}^K \frac{1}{\Gamma(N/2)} \left(\prod_{k=1}^{K} \frac{1}{\sigma_k}\right) 2^{1-\frac{N}{2}} \int_0^\infty \mathrm{d}\rho\, \rho^{K-1} \\ &\times \int_0^\pi \mathrm{d}\vartheta \sin^{K-2}(\vartheta) \sigma_i^2 \rho^2 \cos^2(\vartheta) \left(\sqrt{\frac{N}{T}}\rho\right)^{\frac{N-K}{2}} \\ &\times \mathcal{K}_{\frac{K-N}{2}}\left(\sqrt{\frac{N}{T}}\rho\right) \int \mathrm{d}\Omega_{K-1}\end{aligned} \tag{55}$$

with the surface of the corresponding K-dimensional sphere

$$\int \mathrm{d}\Omega_{K-1} = \frac{2\pi^{(K-1)/2}}{\Gamma((K-1)/2)} \prod_{k=1}^{K} \sigma_k \tag{56}$$

We obtain

$$\mathrm{var}(V_i) = 2\sigma_i^2 \frac{T}{N} \frac{\Gamma(N/2+1)}{\Gamma(N/2)} = \sigma_i^2 T \tag{57}$$

Thus, the variance of every V_i only depends on the standard deviation σ_i and the time T.
