# Supplementary material for: A Random Matrix Approach to Credit Risk
Source: PLoS One. 2014 May 22;9(5):e98030. doi: 10.1371/journal.pone.0098030 (PMC4031172; doi:10.1371/journal.pone.0098030)
Supplement: Appendix S4 — (PDF) [file pone.0098030.s004.pdf]

Appendix S4 Second order approximation of average loss distribution

We rearranged the constants so that each term in $r(\nu, z)$ is normalized to unity. The quantity $r(\nu, z)$ can now be written as

$$r(\nu, z) = \exp\left(\sum_{k=1}^{K} \ln\left[\left(\int_0^{F_k} \mathrm{d}V_k \overbrace{\exp\left(i\nu f_k\left(1 - \frac{V_k}{F_k}\right)\right)}^{q(\nu, F_k)} + \int_{F_k}^{\infty} \mathrm{d}V_k\right)\right.\right.$$
$$\left.\left. \times \frac{\sqrt{N}}{2\sigma_k V_k \sqrt{\pi z T}} \exp\left(-\frac{N(\ln(V_k/V_{k,0}) - (\mu_k - \sigma_k^2/2)T)^2}{4zT\sigma_k^2}\right)\right]\right) \tag{58}$$

We expand $q\left(\nu, F_k\right)$ as the power series

$$q\left(\nu, F_k\right) = \sum_{j=0}^{\infty} \frac{(i\nu f_k)^j}{j!} \left(\frac{F_k - V_k}{F_k}\right)^j \tag{59}$$

Due to the normalization of $\langle p^{(\mathrm{mv})}(V)\rangle$, after insertion into Eq. (58) the non-default term and the integral over first term of Eq. (59) become one. Thus, we can start the sum at $j = 1$ and obtain

$$r(\nu, z) = \exp\left(\sum_{k=1}^{K} \ln\left(1 + \sum_{j=1}^{\infty} \frac{(i\nu f_k)^j}{j!} m_{j,k}(z)\right)\right) \tag{60}$$

with

$$m_{j,k}(z) = \frac{\sqrt{N}}{2\sigma_k \sqrt{\pi z T}} \int_0^{F_k} \frac{1}{V_k} \left(\frac{F_k - V_k}{F_k}\right)^j$$
$$\times \exp\left(-\frac{N(\ln(V_k/V_{k,0}) - (\mu_k - \sigma_k^2/2)T)^2}{4zT\sigma_k^2}\right) \mathrm{d}V_k \tag{61}$$

The integrals in Eq. (61) can be expressed with the generalized hypergeometrical function ${}_p\mathcal{F}_q$. However, the integral representation (61) is more intuitive. Moreover, for explicit $m = 1, 2$ the integrals can be calculated in a closed form, although this results in bulky expressions.

Expanding the logarithm and collecting all terms up to the second order in f_k yields

$$\left\langle p^{(\mathrm{loss})}(L)\right\rangle \approx \frac{1}{2\pi\Gamma(N/2)} \int_0^{\infty} \mathrm{d}z\, z^{\frac{N}{2}-1} \exp(-z) \int_{-\infty}^{+\infty} \mathrm{d}\nu \exp(-i\nu L)$$
$$\times \exp\left(\sum_{k=1}^{K} i\nu m_{1,k}(z) f_k - \frac{\nu^2 f_k^2}{2}(m_{2,k}(z) - m_{1,k}(z)^2)\right) \tag{62}$$
$$= \frac{1}{2\pi\Gamma(N/2)} \int_0^{\infty} \mathrm{d}z\, z^{\frac{N}{2}-1} \exp(-z) \int_{-\infty}^{+\infty} \mathrm{d}\nu$$
$$\times \exp\left(-i\nu L + i\nu \sum_{k=1}^{K} f_k m_{1,k}(z) - \frac{\nu^2}{2} \sum_{k=1}^{K} f_k^2 \left(m_{2,k}(z) - m_{1,k}(z)^2\right)\right)$$

Now we can solve the ν integral leading to Eq. (26).
